# *Impatiens wuyiensis* (Balsaminaceae), a new species from Fujian of Southeast China, based on morphological and molecular evidences

**DOI:** 10.1186/s40529-020-00306-1

**Published:** 2020-11-02

**Authors:** Jian-Sheng Wang, Yi-Fei Lu, Yue-Liang Xu, Shui-Hu Jin, Xiao-Feng Jin

**Affiliations:** 1 School of Forestry and Bio-Technology, Zhejiang Agricultural and Forestry University, Lin’an, 311300 Hangzhou China; 2 College of Life Sciences, Zhejiang University, Hangzhou , 310058 China; 3 Zhejiang Museum of Natural History, Hangzhou, 310014 China; 4 Zhejiang Provincial Key Laboratory for Genetic Improvement and Quality Control of Medicinal Plants & College of Life and Environment Sciences, Hangzhou Normal University, Hangzhou , 311121 China

**Keywords:** Danxia landform, Flora of Southeast China, *Impatiens wuyiensis*, Sect. *impatiens*, Taxonomy

## Abstract

**Background:**

Southeast Asia, together with tropical Africa, Madagascar, South India and Sri Lanka, and the eastern Himalayas, are the five primary hotspots of species diversity of *Impatiens* (Balsaminaceae). China is also rich in *Impatiens* species, especially in the limestone karsts or ‘Danxia’ landforms. With zygomorphic flowers and diverse corolla morphology and color, the species in *Impatiens* are well-known for their ornamental use, but they are also notorious in taxonomy. During the preparation of revision of *Impatiens* in Zhejiang and adjacent regions, an unknown species was collected from Mt. Wuyi in Fujian Province, Southeast China.

**Results:**

Phylogenetic analyses based on nuclear ITS, chloroplast *atp*B-*rbc*L and *trn*L-F sequences, together with micromorphology of pollen grains and seed coats, strongly supported the close relationship of the new species with *Impatiens platysepala* Y.L.Chen and *I. chloroxantha* Y.L.Chen. In turn, both molecular data and morphological characters also were sufficient to distinguish the new species from the other two counterparts.

**Conclusions:**

Our detailed morphological observations and molecular phylogenetic analyses support the recognition of *Impatiens wuyiensis* as a species new to science.

## Background

*Impatiens* L., containing more than 1000 species, is one of the largest genera of seed plants and is a notorious genus for taxonomic difficulty (Hooker 1908; Chen 1978; Yu et al. 2016). The genus *Impatiens* has zygomorphic flowers with great diversity both in corolla color and morphology, thus it was regarded as ‘the dicot counterpart of the orchid’ (Yuan et al. 2004; Yu et al. 2016). Some species, e.g. *Impatiens balsamina* L. (‘balsam’) and *I. walleriana* Hook.f. (‘busy lizzie’), are well-known for horticultural value and widely cultivated in East to South China. The genus *Impatiens* differs from its sister group *Hydrocera* Blume ex Wight & Arn., a monotypic genus, in having lateral petals (sometimes also called wings) united in pairs and valvate and explosive capsules (Chen 2001; Chen et al. 2007). With membranous dried flowers and easily dehiscent fruits on specimens, descriptions of floral and fruit characters on herbarium specimens may therefore be incomplete or ambiguous. Consequently, field investigations are essential for accurately describing the reproductive characters (Chen 1978; Yu 2012).

The genus *Impatiens* has five recognized diversity hotspots: viz. tropical Africa, Madagascar, South India and Sri Lanka, the eastern Himalayas, and Southeast Asia (Song et al. 2003; Yuan et al. 2004). China is also rich in *Impatiens* species, including over 250 species which are mainly distributed in south-western regions, especially in Yunnan, Sichuan, Guangxi, Tibet and Guizhou (Chen 1978; Chen et al. 2007; Shui et al. 2011). Recently, molecular phylogenetics has contributed greatly to the understanding of relationships within *Impatiens* (Yuan et al., 2004; Janssens et al. 2006, 2007, 2012; Yu et al. 2016). Moreover, micromorphology of pollen grains and seed coats of *Impatiens* have great taxonomic values (Lu 1991; Lu and Chen 1991; Song et al. 2005; Janssens et al. 2012; Cai et al. 2013), and systematic implications (Jin et al. 2008; Guo et al. 2016; Zeng et al. 2016).

Chen (1988, 1989, 1999) carried out the taxonomic studies and described eight new species from Zhejiang, Anhui and Jiangxi provinces. However, the previous collection of this area was not satisfactory till now. Our field surveys of *Impatiens* from Zhejiang and adjacent regions in East China have resulted in the discovery of several new species and some infraspecific taxa (*Impatiens taishunensis*, Chen and Xu 1993; *I. jiulongshanica*, *I. neglacta*, *I. suichangensis* and *I. tienmushanica* var. *longicalculata*, Xu and Chen 1999; *I. huangyanensis*, Jin and Ding 2002; *I platysepala* var. *kuocangshanica*, Jin and Zhang 2003; *I. yilingiana* and *I. huangyanensis* subsp. *attenuata*, Jin et al. 2008; *I. chekiangensis* var. *multiflora* and var. *cangnanensis*, Xu et al. 2018). In this study we report an additional distinctive new species, *I. wuyiensis*, from one of the typical Danxia landforms in northern Fujian Province, China.

Danxia, literally meaning ‘rosy cloud’, is a type of sandstone landform found in southeastern, southwestern and northwestern China that consist of a red bedrock characterized by steep cliffs, isolated peaks, steep pillars, ravines, mountains and hills that have formed after a long period of erosion by wind and running water (Liu et al. 1999; Peng et al. 2018). The Danxia landform in Fujian and Guangdong Provinces of China shows the block mountains and looks like isolated islands (Peng et al. 2018).

Based on its flower structure, and micromorphology of pollen grains and seeds, *Impatiens wuyiensis* is similar to *I. platysepala* and *I. chloroxantha*, but evidences of molecular data (ITS, *trn*L-F and *atp*B-*rbc*L) and other morphological characters well support the status of this taxon as new.

## Methods

### SEM observations on pollens and seeds

Dried mature pollen grains and seeds of the new species, together with its allied *Impatiens platysepala* and *I. chloroxantha*, were directly collected from the field (see further methodological details in Appendix 1). Pollen grains and seeds were mounted on stubs using double-sided adhesive tape, and directly coated with a layer of gold. The coated pollen grains and seeds were observed and photographed under a Hitachi SU8010 SEM. The sizes of pollen grains and seeds were respectively measured on twenty grains per species using light microscopy. Pollen terminology follows Wang and Wang (1983) and Lu (1991), and that of seed follows Liu et al. (2004) and Lu and Chen (1991).

### Taxon sampling and DNA sequencing

Seventeen samples representing eight species and one variety of *Impateins* were collected, and total genomic DNA of these samples were isolated from dried leaf tissue using the TIANGEN Plant Genomic DNA Kit (China). Three DNA regions, ITS (White et al. 1990), *atp*B*-rbc*L (Janssens et al. 2006), and *trn*L*-*F (Taberlet et al. 1991) were amplified. PCR (the polymerase chain reaction) mixture (25µL) contained 12.5 μL 2 × Reaction Mix, 1.0 μL each primer (10 pmol/µL), 1.0 μL genomic DNA (< 0.5 μg), 0.5 μL Golden DNA Polymerase (2.5 U/μL) and 9.2 μL ddH2O. The PCR conditions were: 1 cycle of 3 min at 94 °C for denaturation, 35 cycles of 30 s at 94 °C for denaturation (for ITS, 38 cycles), 30 s at 55 °C for annealing and 1 min at 72 °C for extension (for ITS, 45 s), with a final 5 min extension at 72 °C. The successful PCR products were checked using a 1% agarose gel electrophoresis. Sequencing was carried out on an ABI 3730 automated sequencer (Applied Biosystems, USA).

A total of 116 sequences representing 34 taxa (*Impatiens* species and varieties, plus *Hydrocera triflora* as outgroup) were used to test the phylogenetic placement of the new species. The sampling representatively covered all the sections (with the exception of sect. *Tuberosae*) known in the phylogeny of *Impatiens* (Yu et al. 2016). Fifty-one sequences representing nine taxa being collected in the field were newly generated in this study. The rest of the sequences were obtained from GenBank (Appendix 1).

### Phylogenetic analysis

Sequences were assembled and edited using DNAStar Lasergene 7.1 software. Assembled sequences of the same locus were aligned using MAFFT online version (https://mafft.cbrc.jp/alignment/server/) and trimmed manually in Mega 7.0 (Kumar et al. 2016). The best-fit nucleotide substitution models were determined by the Akaike Information Criterion (AIC) using JmodelTest 2 (Darriba et al. 2012), which resulted in ITS for GTR + I + G, *atp*B-*rbc*L for GTR + G and *trn*L-F for GTR + G. Bayesian Inference (BI) was conducted in MrBayes 3.2.6 (Ronquist and Huelsenbeck 2003), running for 10 million generations and sampling one tree every 1000 generations with four chains of Markov chain Monte Carlo (MCMC). A 50% majority-rule consensus tree was calculated after discarding the first 25% trees as burn-in. A Maximum parsimony (MP) analysis was conducted using PAUP v.4.0b10 (Swofford 2003), using a heuristic search algorithm with 1000 random addition replicates and tree bisection-reconnection (TBR) branch-swapping. Node support was assessed using 1000 MP bootstrap (BS) replicates.

## Results and discussion

**Fig. 1:**
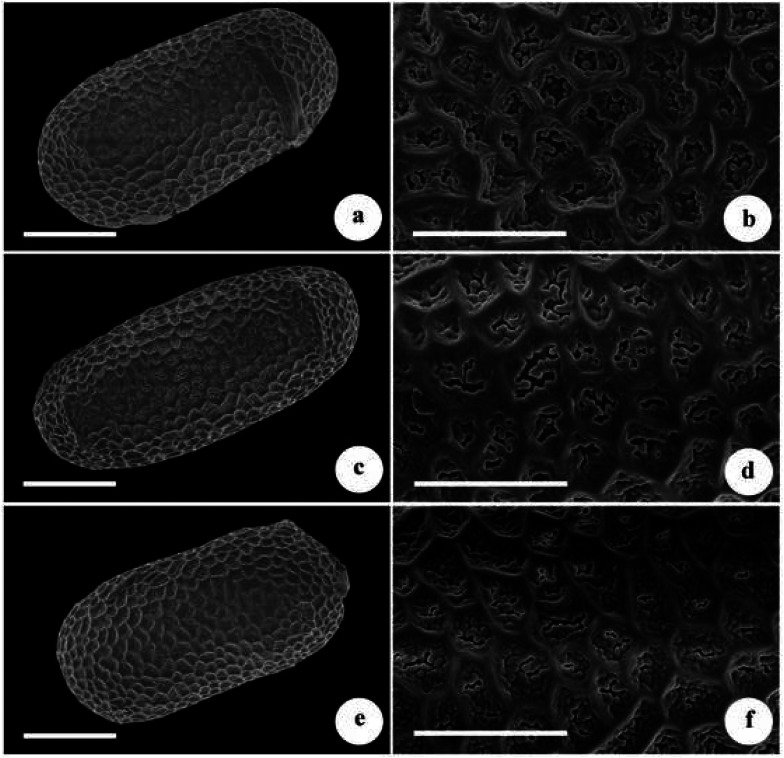
Micromorphology of pollen grains of *Impatiens wuyiensis*, *I. platysepala* and *I. chloroxantha*. **a, b** *I. wuyiensis*; **c, d** *I. platysepala*; **e, f** *I. chloroxantha*. **a, c, e** overview of pollen grain (scale bar: 10 μm); **b, d, f** sexine sculpture (scale bar: 5 μm)

Micromorphology of the pollen grains of the new species, *Impatiens platysepala* and *I. chloroxantha* were shown in Fig. 1. All were oblong-ellipsoid, regularly reticulate, densely granulate in lumen, and with a few perforations. The size of the pollen grains of the new species (E1 × E2) was 35.20 (± 1.01) × 16.27 (± 1.00) µm, and those of *I. platysepala* and *I. chloroxantha* were respectively 35.27 (± 1.30) × 16.98 (± 1.21) µm and 34.08 (± 1.82) × 16.40 (± 1.26) µm. This confirmed that the size and micromorphology of pollen grains is similar among three species (Fig. 1).

**Fig. 2:**
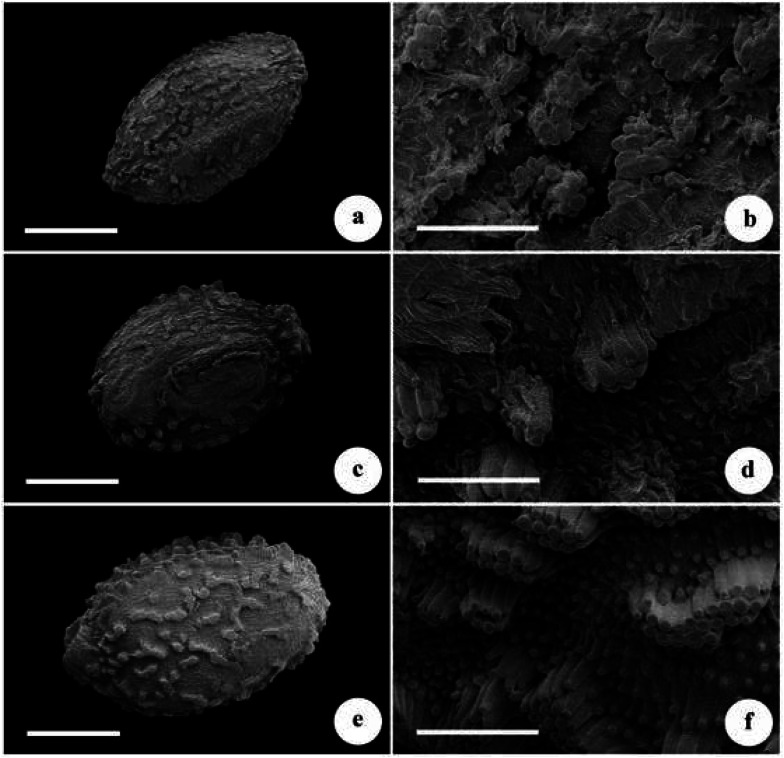
Micromorphology of seeds of *Impatiens wuyiensis*, *I. platysepala* and *I. chloroxantha*. **a, b** *I. wuyiensis*; **c, d** *I. platysepala*; **e, f** *I. chloroxantha*. **a, c, e** shape of seeds (scale bar: 1 mm); **b, d, f** surface ornamentation of seedcoat (scale bar: 200 μm)

Seeds of the new species, *Impatiens platysepala* and *I. chloroxantha* were all ovoid, slightly compressed, and surface cells regularly elevated, with granules on cell surfaces. The micromorphology of seed coat was revealed to be closely similar in the three studied species (Fig. 2). The seed size of the new species (length × width) was 2.39 (± 0.12) × 1.29 (± 0.12) mm, and those of *I. platysepala* and *I. chloroxantha* were respectively 2.51 (± 0.10) × 1.54 (± 0.11) mm and 2.96 (± 0.20) × 1.63 (± 0.16). Observed seed size of *I. chloroxantha* was larger than in *I. platysepala* and the new species (Fig. 2).

**Fig. 3:**
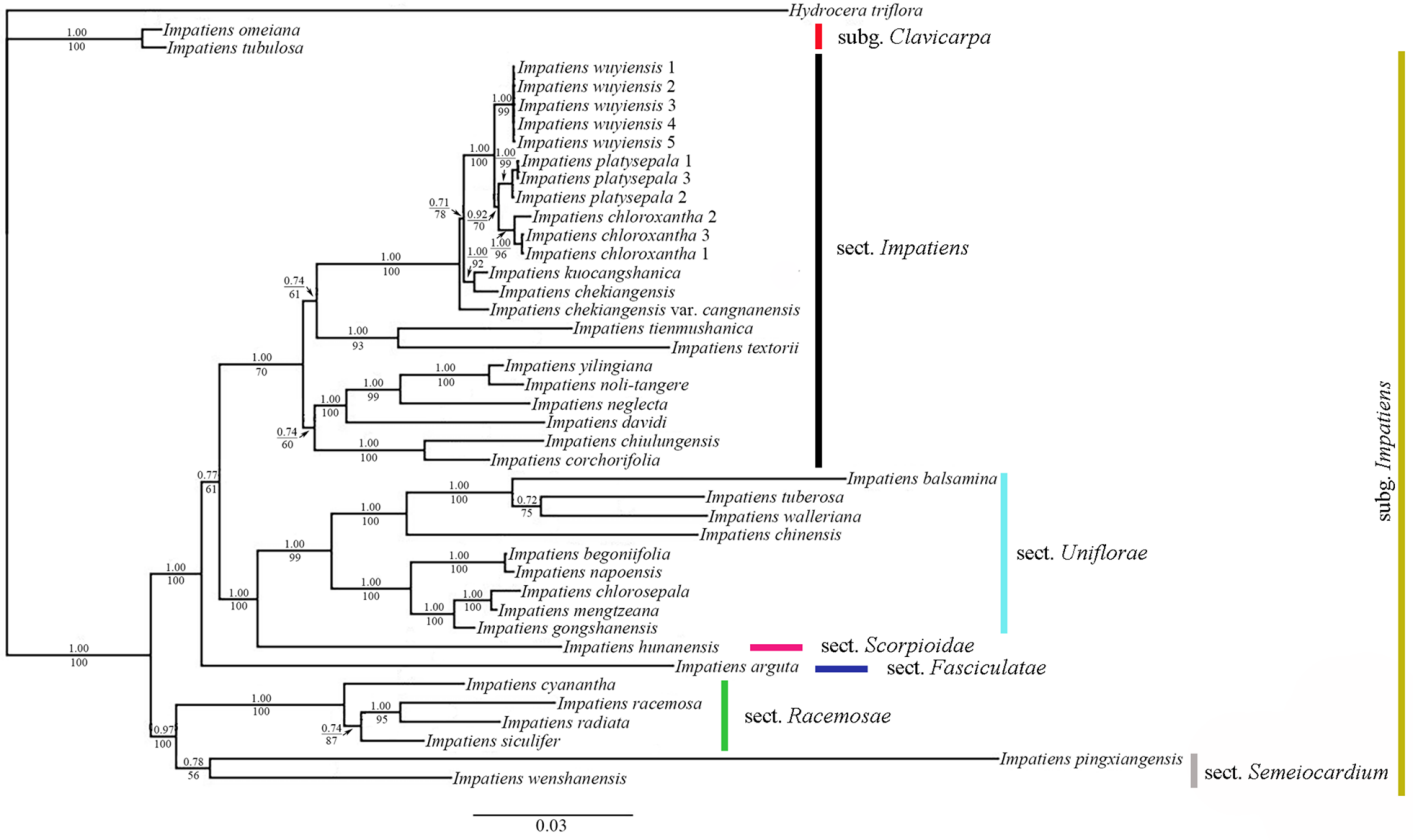
Majority consensus tree inferred from Bayesian analysis based on the nrDNA ITS and cpDNA *atp*B-*rbc*L and *trn*L-F data. Values above the branches are Bayesian posterior probabilities (PP ≥ 50%), and those below the branches are maximum parsimony bootstrap supports (BS ≥ 50%)

The combined dataset of ITS, *atp*B-*rbc*L and *trn*L-F included 2505 aligned characters (ITS: 697 bps, *atp*B-*rbc*L: 869 bps and *trn*L-F: 939 bps). The phylogram with posterior probability values (PP) of Bayesian analysis and bootstrap supports (BS) of Maximum parsimony analysis is respectively depicted in Fig. 3. Five individuals of the new species were shown forming a well-supported clade (PP = 1.00, and BS = 99), which was sister to the clade of two similar species, *I. platysepala* and *I. chloroxantha*. Together with the other ten speices (*I. chekiangensis*, *I. noli-tangere*, *I. tienmushanica*, *I. davidi* etc.), these species are easily distiguished as members in *I.* sect. *Impatiens*, which is characterized by inflorescence shortly racemose or umbel-like, ovary 5-capellate, and capsules clavate (Yu et al. 2016).

## Conclusion

According to our results, we proceed to formally describe the new species.

**Fig. 4:**
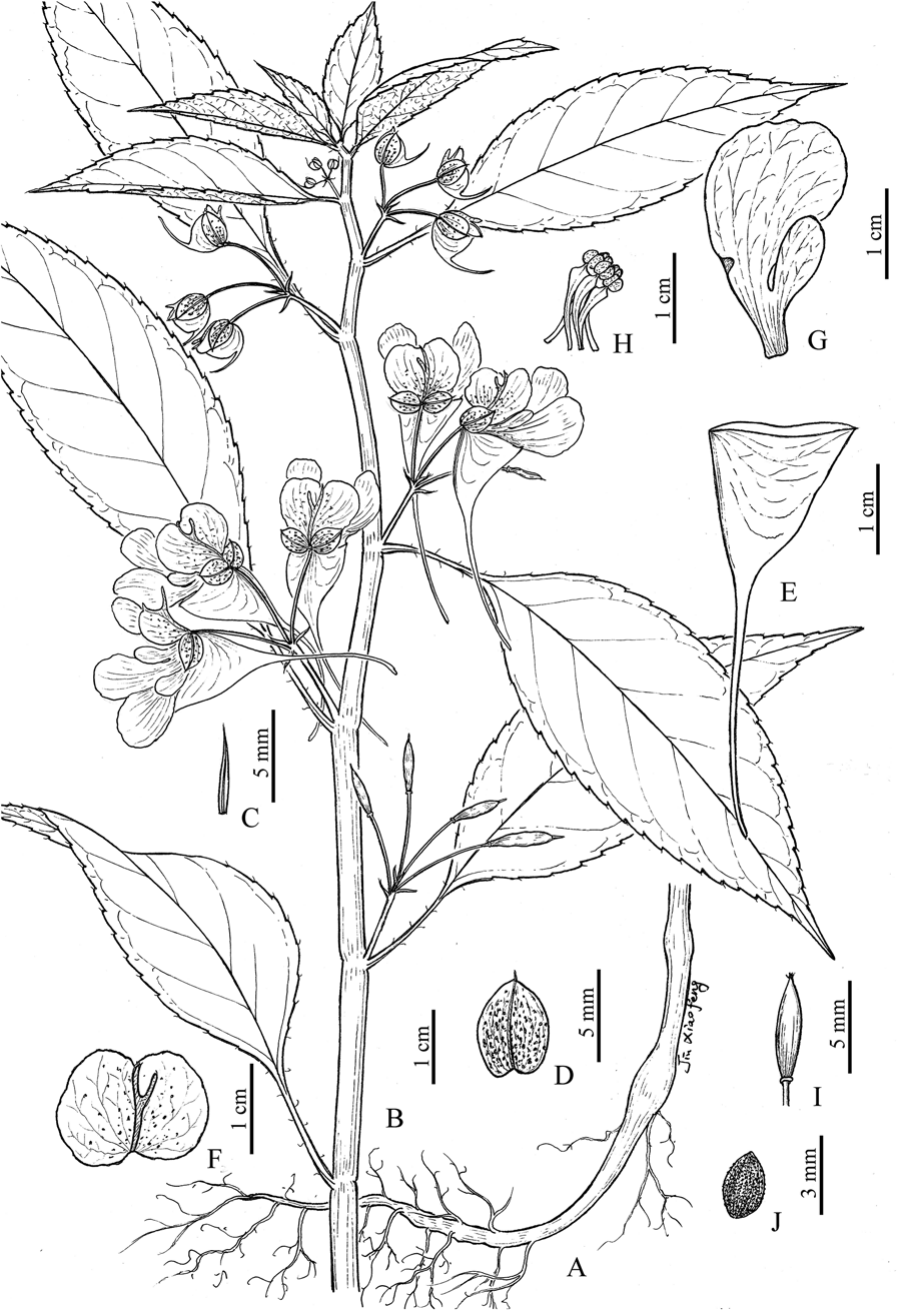
*Impatiens wuyiensis* J.S.Wang, Y.F.Lu & X.F.Jin. **a** habit (showing lower part and roots); **b** habit (showing upper part); **c** bract; **d** lateral sepal; **e** lower sepal; **f** upper petal; **g** lateral petals; **h** stamens; **i** ovary; **j** seed (Drawn by X.F. Jin from the holotype)

**Fig. 5:**
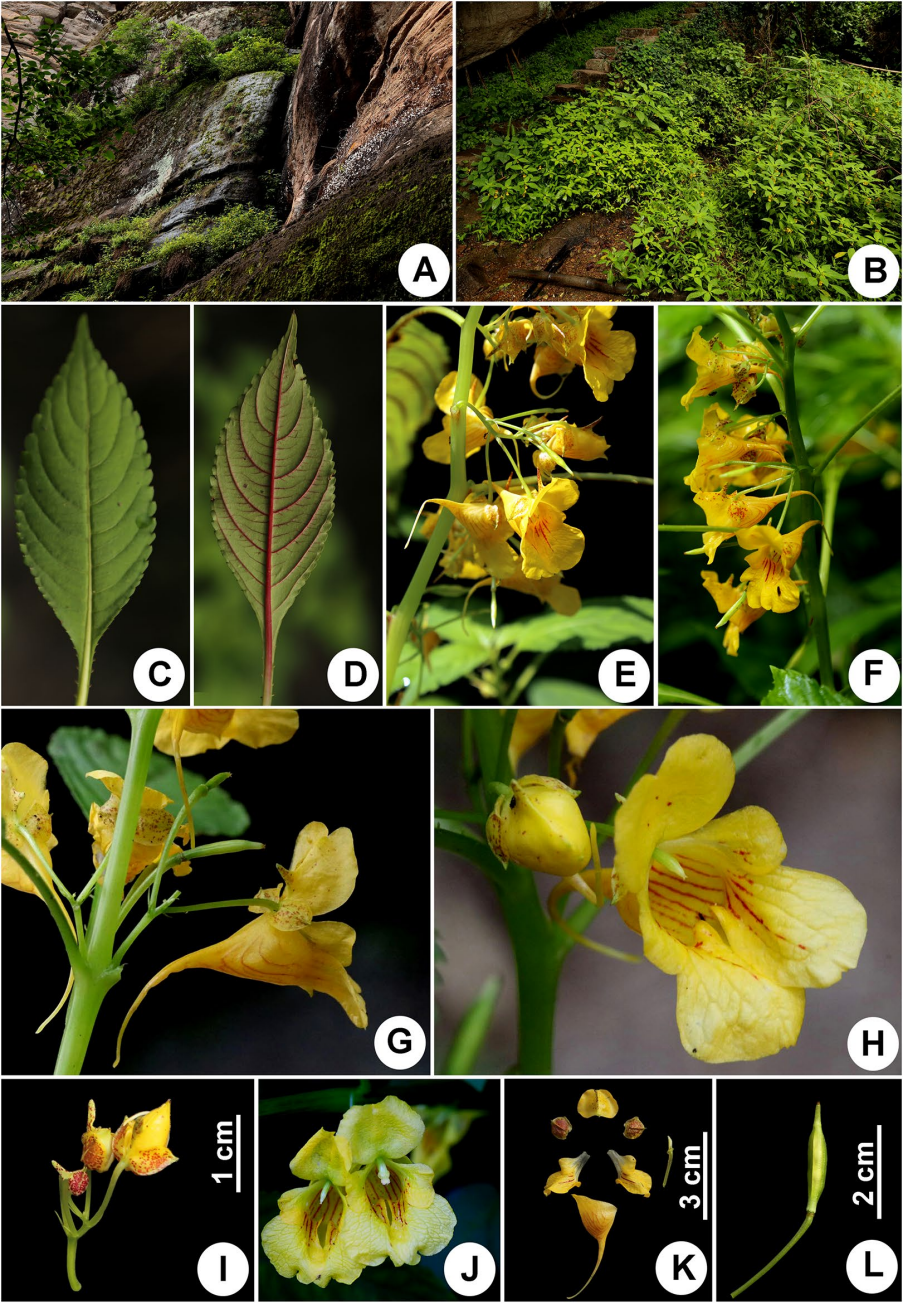
*Impatiens wuyiensis* J.S.Wang, Y.F.Lu & X.F.Jin. **a, b** habitat; **c** adaxial surface of leaf; **d** abaxial surface of leaf; **e**, **f** inflorescence; **g** lateral view of flower; **h** frontal view of flower; **i** pedicel and bract; **j** upper petal and lateral united petals; **k** flower structure; **l** capsule

**Impatiens wuyiensis** J.S.Wang, Y.F.Lu & X.F.Jin, **sp. nov.**: TYPE: CHINA, Fujian, Wuyishan City, Mt. Wuyi, Dawangfeng, moist places by roadside, 27°38′57.13′’N, 117°57′47.42′’E, alt. 420 m, 23 May 2018, *X.F. Jin, Y.F. Lu & J.S. Wang 4158* (holotype: ZM barcode ZMNH0068001, isotypes: HTC barcode HTC0021906, KUN, PE, ZJFC). 武夷凤仙花 (Figs. 4 and 5).

**Diagnosis:** *Haec species in characteribus floralibus est affinis Impatienti platysepalae Y.L. Chen et I. chloroxanthae Y.L. Chen, a qua floribus luteis, sepalis lateralibus dense purpureo-rubro-maculatis, costas vexilli dorso in medio breviter clavatis vel cornutis, bracteis herbaceis linearis vel anguste ovato-lanceolatis, 3–4 mm longis, ca. 0.5 mm latis differt.*

**Description:** Herbs annual, 20–75 cm tall, glabrous. Stems succulent, erect, usually simplex; lower nodes swollen or slightly swollen. Leaves alternate; blades membranous, ovate-elliptic or elliptic-oblong, rarely ovate or oblong, 2.5–13 cm long, 1.5–5.5 cm wide, apex acuminate, base cuneate and gradually attenuate into a 1–8 cm long petiole (upper aggregated leaves subsessile), margin crenate and mucronulate, dark green adaxially, pale green and pale red abaxially, lateral veins 6–8 pairs, obliquely curved. Inflorescence in leaf axils, shortly racemose or umbel-like, shorter than leaves; peduncles shorter than petioles, 10–13 mm long, 2–4-flowered, rarely 5- or 6-flowered; pedicels 14–20 mm long, base bracteate; bracts herbaceous, persistent, linear or narrowly ovate-lanceolate, 3–4 mm long, ca. 0.5 mm wide, apex acuminate. Flowers golden yellow, 4–5 cm long, 2–2.2 cm wide. Lateral sepals 2, broadly ovate or ovate-rounded, 6–7 mm long, 5.5–6.5 mm wide, densely purple-red spotted, base nearly aequlilateral, apex obtuse and mucronulate, abaxial midvein keeled. Lower sepal funnelform, 4–4.5 cm long, purple-red striate, base abruptly narrowed into a 2.5–3 cm long and slightly incurved spur; mouth vertical, 16–18 mm wide, tip acute. Upper petal orbicular, 11–13 mm long, 19–21 mm wide, purple-red spotted, apex emarginate and mucronate, abaxial midvein keeled and clavate or corned at middle. Lateral united petals 22–25 mm long, 12–14 mm wide, purple-red striate, 2-lobed; basal lobes oblong, 8–9 mm long, 3–3.5 mm wide, apex obtuse, with a 4–4.5 mm long stipe; distal lobes dolabriform, apex obtuse, with reflexed and golden yellow auricle. Stamens 5; filaments 4–6 mm long; anthers ovoid, apex obtuse. Ovary fusiform, ca. 3 mm long, apex acute. Capsule clavate, 10–20 mm long, apex with a 2–3.5 mm long beak. Seeds ovoid, brown, ca. 2.5 mm long, ca. 2 mm wide, densely verrucous.

**Distribution, habitat and ecology:** *Impatiens wuyiensis* seems to be restricted to Mt. Wuyi. It is widely distributed along several scenic spots, such as Dawangfeng, Tianyoufeng, Shuiliandong and Lianhuafeng views. It grows in moist places by roadside or in grasslands, on rock face, under forest or at forest margins, at an elevation between 220 and 430 m (Fig. 5: a, b).

**Phenology:** Flowering and fruiting of this new species is from early April to late September.

**Etymology:** The specific epithet ‘*wuyiensis*’ is derived from the type locality, Mt. Wuyi, northern Fujian Province.

**Fig. 6:**
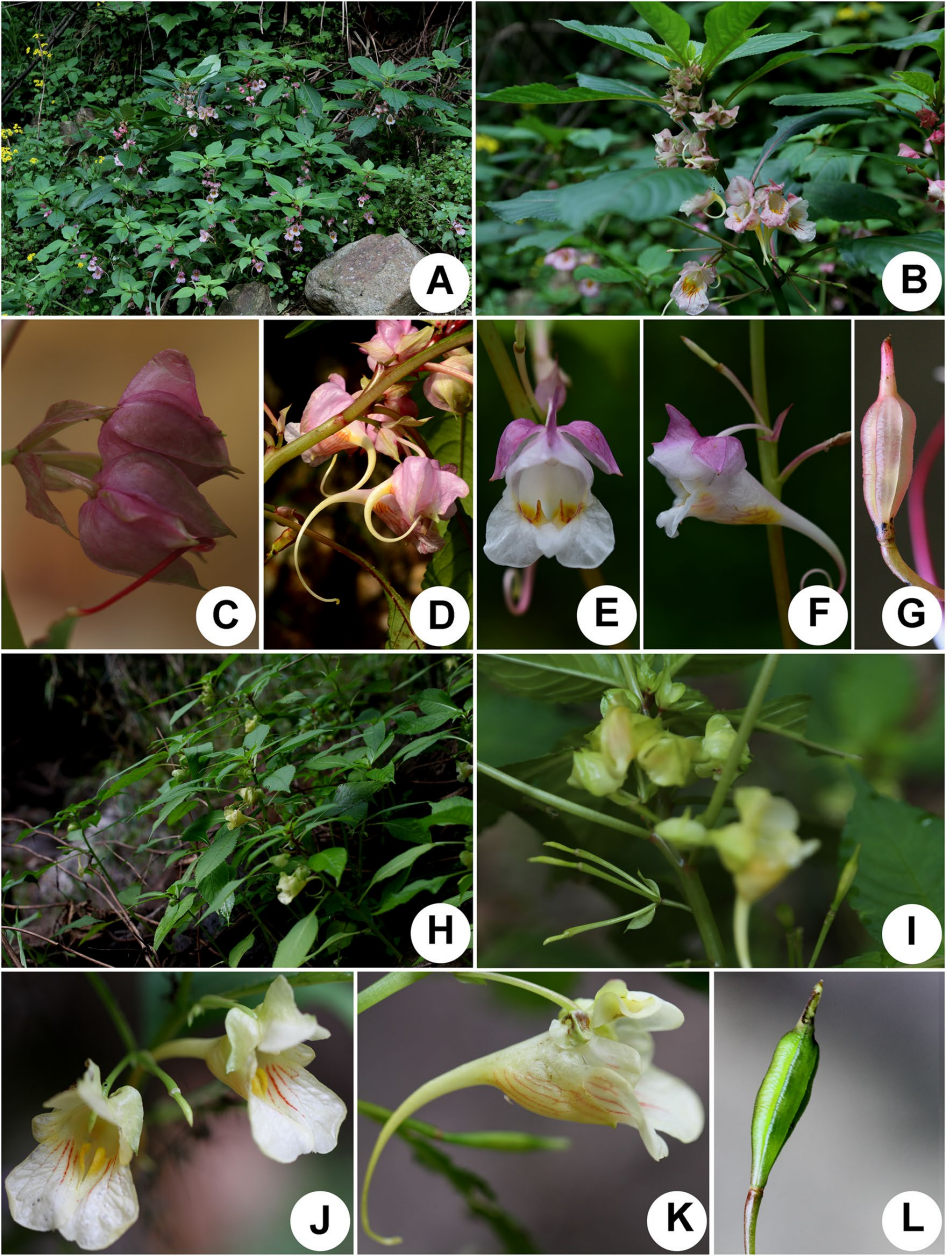
*Impatiens platysepala* Y.L.Chen (**a‒g**) and *I. chloroxantha* Y.L.Chen (**h‒l**). **a** habitat; **b** upper part of plant; **c** bracts and flowers; **d** inflorescence; **e** frontal view of flower; **f** lateral view of flower; **g** capsule; **h** habitat; **i** inflorescence and bracts; **j** frontal view of flower; **k** lateral view of flower; **l** capsule

**Notes:** This new species is morphologically similar to *Impatiens platysepala* and *I. chloroxantha* in habit and floral structure (Chen 1988, 2001; Zhang et al. 2017), but is easily distinguished from these two species in having flowers golden yellow, lateral sepals densely purple-red spotted, upper petals with abaxial midvein clavate or corned at midle, bracts herbaceous, linear or narrowly ovate-lanceolate, 3–4 mm long, ca. 0.5 mm wide. Morphological characters distinguishing the new species from *Impatiens platysepala* and *I. chloroxantha* are detailed in Table 1, Figs. 5 and  6.

**Table 1:** Comparison of *Impatiens wuyiensis*, *I. platysepala* and *I. chloroxantha*

Characters	*Impatiens wuyiensis*	*Impatiens platysepala*	*Impatiens chloroxantha*
Leaves			
Abaxial color	Pale green and pale red	Pale green, rarely pale red	Pale green
Lateral veins	6–8 pairs	8–11 pairs	7–9 pairs
Bracts			
Texture	Herbaceous	Thinly membranous	Thinly membranous
Shape	Linear or narrowly ovate-lanceolate	Ovate-lanceolate	Ovate or narrowly ovate
Size	3–4 × ca. 0.5 mm	10–12 × 4–6 mm	8–11 × 4–5 mm
Flower			
Color	Golden yellow	Pink	Yellow-green
Lateral sepals	Densely purple-red spotted	Not spotted	Not spotted
Upper petals	Middle with abaxial midvein clavate or corned	Middle with abaxial midvein cristate	Middle with abaxial midvein cristate

Micromorphology of pollen grains and seeds of the new species, *Impatiens platysepala* and *I. chloroxantha* revealed that these three species are closely related. Phylogenetic analysis detected that the new species is also related to *I. platysepala* and *I. chloroxantha*, but considerably different from each another with strong supports.

Additional specimens examined (paratypes): Fujian, Wuyishan City, Mt. Wuyi, Dawangfeng, moist places by roadside, 27°38′57.15′’N, 117°57′47.36′’E, alt. 430 m, 23 May 2018, *X.F. Jin, Y.F. Lu & J.S. Wang 4156* (ZJFC, ZM), ibid., 23 May 2018, *X.F. Jin, Y.F. Lu & J.S. Wang 4157* (PE, ZJFC, ZM); Mt. Wuyi, Shuiliandong, in grass under forest, 27°40′56.23′’N, 117°58′32.04′’E, alt. 220 m, *X.F. Jin, Y.F. Lu & J.S. Wang 4165* (ZM), *4166* (KUN, PE, ZM), *4167* (KUN, PE, ZM), ibid., by stream, alt. 270 m, 4 April 2019, *X.F. Jin, Y.F. Lu & J.S. Wang 4375* (ZJFC, ZM), ibid., 21 September 2019, *X.F. Jin & Y.F. Lu* s. n. (ZM); Mt. Wuyi, Tianyoufeng, in grass by roadside, alt. 227 m, 7 May 2019, *Y.F. Lu 186* (ZM).

## Data Availability

All DNA sequences generated in this study have been registered to GenBank.

## Appendix 1

***Taxon***: NCBI accession numbers (ITS/*atp*B-*rbc*L/*trn*L-F), voucher information. Samples marked with ‘*’ were newly added, and ‘#’ were those pollen grains and seeds collected.

*Hydrocera triflora* (L.) Wright & Arn.: AY348853/ DQ147895/ -, Sri Lanka, *Robyns 7260, 0,249,369* (BR).

*Impatiens arguta* Hook.f. & Thomson: AY348746/ DQ147812/ KP776116, China, Yunnan and Xizang, *Y. M. Yuan CN2k1-74* (NEU) and *S.X. Yu 5406* (PE).

*I. balsamina* L.: AY348749/ DQ147816/ -, Kruidtuin Leuven (Cult.), *Janssens SJ 001* (LV).

*I. begoniifolia* S. Akiyama & H. Obha: AY348752/ DQ147819/ -, China, Yunnan, *Y. M. Yuan CN2k1-51* (NEU).

*I. chekiangensis* Y.L.Chen: KP776064/ KP776014/ KP776121, China, Zhejiang, *H.N. Qin *et al*. 20,141* (PE).

*I. chekiangensis* Y.L.Chen var. *cangnanensis* Y.L.Xu & X.F.Jin*: MN974564/ MN974547/ MN974581, China, Zhejiang, Cangnan, Dangyuanlin, *Y.L Xu 318* (ZM).

*I. chinensis* L.: AY348761/ DQ147825/ KP776122, China, Yunnan and Guangxi, *Y.M. Yuan CN2k1-49* (NEU) and *I. chiulungensis* Y.L.Chen: KP776066/ KP776016/ KP776124, China, Sichuan, *S.X. Yu *et al*. 3989* (PE).

*I. chlorosepala* Hand.-Mazz.: KP776067/ KP776017/ -, China, Guangxi, *S.X. Yu *et al*. 4209* (PE).

*I. chloroxantha* Y.L.Chen (individual 1)*: MN974563/ MN974546/ MN974580, China, Zhejiang, Suichang, Mt. Jiulong, Luohanyuan, *X.F. Jin s. n.* (HTC). *I. chloroxantha* Y.L.Chen (individual 2)*#: MN974573/ MN974556/ MN974590, China, Zhejiang, Suichang, Mt. Jiulong, Luohanyuan, *J.L. Liu s. n.* (HTC). *I. chloroxantha* Y.L.Chen (individual 3)*: MN974574/ MN974557/ MN974591, China, Zhejiang, Suichang, Mt. Jiulong, Luohanyuan, *J.L. Liu s. n.* (HTC).

*I. corchorifolia* Franch.: AY348767/ DQ147831/ KP776127, China, Yunnan and Sichuan, *Chassot & Y.M. Yuan 99–173* (NEU) and *S.X. Yu *et al*. 4596* (PE).

*I. cyanatha* Hook.f.: AY348770/ DQ147833/ -, China, Yunnan, *Y.M. Yuan CN2k1-84* (NEU).

*I davidi* Franch.*: MN974568/ MN974551/ MN974585, China, Zhejiang, Yinzhou, Wulongtan, *X.F. Jin & Y.F. Lu 4357* (HTC).

*I. gongshanensis* Y.L.Chen: KP776074/ KP776024/ KP776135, Myanmar, Putao, *PT-ET 975* (PE).

*I. hunanensis* Y.L.Chen: KP776077/ KP776028/ KP776137, China, Guangxi, *S.X. Yu 3759* (PE).

*I. kuocangshanica* (X.F.Jin & F.G.Zhang) X.F.Jin & Y.L.Xu*: MN974567/ MN974550/ MN974584, China, Zhejiang, Linhai, Mt. Kuocang, Zhangjiadu, *B.Y. Ding & X.F. Jin 6924* (PE).

*I. mengtzeana* Hook.f.: AY348806/ DQ147858/ -, China, Yunnan, *Y.M. Yuan CN2k1-60* (NEU).

*I. neglecta* Y.L.Xu & Y.L.Chen*: MN974569/ MN974552/ MN974586, China, Zhejiang, Longquan, Mt. Fengyang, *Y.F. Lu 203* (HTC).

*I. napoensis* Y.L.Chen: AY348811/ DQ147861/ KP776146, China, Yunnan and Guangxi, *Y.M. Yuan CN2k1-61* (NEU) and *S.X. Yu 3049* (PE).

*I. noli-tangere* L.: KP776088/ KP776039/ KP776148, China, Guangxi, *S.X. Yu 4017* (PE).

*I. omeiana* Hook.f.: KP776092/ DQ147864/ KP776152, China, Sichuan and Univ. California Bot. Gard. (Cult.), *S.X. Yu 4093* (PE) and 2002.0214 (UC).

*I. pingxiangensis* H.Y.Bi & S.X.Yu: KP776093/ KP776043/ -, China, Guangxi, *S.X. Yu *et al*. 3088* (PE).

*I. platysepala* Y.L.Chen (individual 1)*#: MN974572/ MN974555/ MN974589, China, Zhejiang, Jiangshan, Zhoucun, *Z.H. Chen *et al*. JS19061201* (HTC). *I. platysepala* Y.L.Chen (individual 2)*: MN974570/ MN974553/ MN974587, China, Zhejiang, Jiangshan, Zhoucun, *Z.H. Chen *et al*. JS19061203* (HTC). *I. platysepala* Y.L.Chen (individual 3)*: MN974571/ MN974554/ MN974588, China, Zhejiang, Jiangshan, Nianbadou, *Z.H. Chen *et al*. JS19061205* (HTC).

*I. racemosa* DC.: KP776098/ DQ147873/ KP776159, China, Yunnan and Xizang, *S.X. Yu 4221* (PE) and *De Haas 2620* (U).

*I. radiata* Hook.f.: AY348824/ KP776047/ KP776160, China, Xizang and Sichuan, *De Haas 2620* (U) and *S.X. Yu *et al*. 4760* (PE).

*I. siculifer* Hook.f.: KP776101/ KP776049/ -, China, Guangxi, *S.X. Yu 3211* (PE).

*I. textori* Miq.: AY348841/ -/ KP776168, Japan and Korea, *Kanno *et al*. 1114* (TUS) and *H.N. Qin *et al*. 18,048* (PE).

*I. tienmushanica* Y.L.Chen*: MN974565/ MN974548/ MN974582, China, Zhejiang, Pan’an, Mt. Dapan, Huaxi, *Y.F. Lu s. n.* (HTC).

*I. tuberosa* H. Perrier: AY348844/ DQ147886/ -, Madagascan origin: Bot. Gard. Univ. Kopenhagen (Cult.), *Janssens SJ 005* (LV).

*I. tubulosa* Hemsl.: KP776108/ KP776056/ KP776172, China, Guangxi, *S.X. Yu 3762* (PE).

*I. walleriana* Hook.f.: AY348849/ DQ147892/ AB043641, Kenya, Nat. Bot. Gard. Meise (Cult.) and Tokyo (Cult.), *O. Phaehler & M. Schnell I08* (NEU), *S3926* (BR) and *H. Fujihashi 3* (TI).

*I. wenshanensis* S.H.Huang: KP776110/ KP776057/ KP776175, China, Guangxi, *S.X. Yu 4044* (PE).

*I. wuyiensis* J.S.Wang, Y.F.Lu & X.F.Jin (individual 1)*: MN974558/ MN974541/ MN974575, China, Fujian, Mt. Wuyi, Shuiliandong, *X.F. Jin *et al*. 4167* (ZM). *I. wuyiensis* J.S.Wang, Y.F.Lu & X.F.Jin (individual 2)*: MN974559/ MN974542/ MN974576, China, Fujian, Mt. Wuyi, Dawangfeng, *X.F. Jin *et al*. 4156* (ZM). *I. wuyiensis* J.S.Wang, Y.F.Lu & X.F.Jin (individual 3)*#: MN974560/ MN974543/ MN974577, China, Fujian, Mt. Wuyi, Dawangfeng, *X.F. Jin *et al*. 4158* (ZM). *I. wuyiensis* J.S.Wang, Y.F.Lu & X.F.Jin (individual 4)*: MN974561/ MN974544/ MN974578, China, Fujian, Mt. Wuyi, Shuiliandong, *X.F. Jin *et al*. 4165* (ZM). *I. wuyiensis* J.S.Wang, Y.F.Lu & X.F.Jin (individual 5)*: MN974562/ MN974545/ MN974579, China, Fujian, Mt. Wuyi, Tianyoufeng, *Y.F. Lu 186* (ZM).

*I. yilingiana* X.F.Jin, S.Z.Yang & L.Qian*: MN974566/ MN974549/ MN974583, China, Zhejiang, Lin’an, Mt. Tianmu, Dashuwang, *X.F. Jin 1901* (HTC).
